# Methodology for Selecting Stable UAV-Based Vegetation Indices for Prediction of Agronomic Variables in Maize Using a Multispectral Sensor

**DOI:** 10.3390/plants15121782

**Published:** 2026-06-09

**Authors:** Charleston dos Santos Lima, Ana Júlia Teixeira Soares, Bárbara da Silva Nogueira, André Luis Vian, Ivan Ricardo Carvalho, Christian Bredemeier

**Affiliations:** 1 Department of Crop Science, Federal University of Rio Grande do Sul (UFRGS), Porto Alegre 91501-970, RS, Brazil; anajuliatex3@gmail.com (A.J.T.S.); barbara.nogueira012@gmail.com (B.d.S.N.); andre.vian@ufrgs.br (A.L.V.); 2 EMBRAPII Center for Embedded Devices and Research in Digital Agriculture (CEDRA)—Senai-RS, São Leopoldo 93025-753, RS, Brazil; 3 Department of Plant Breeding, Regional University of the Northwestern State of Rio Grande do Sul (UNIJUI), Ijuí 98700-000, RS, Brazil; ivan.carvalho@unijui.edu.br

**Keywords:** drone, phenotyping, environment, remote sensing

## Abstract

Plant phenotyping based on unmanned aerial vehicles still faces challenges regarding the direct correlation between spectral information with field-collected variables, due to the influence of environmental factors and the considerable variation among maize phenological stages. Therefore, the objectives of this research were: I) to evaluate the interaction of nitrogen doses and evaluation environments (phenological stages and growing seasons) and variance components for field variables and vegetation indices; II) to identify the most suitable indices according to the evaluation environments; and III) to predict field variables based on relevant vegetation indices identified through the proposed methodology. The study was conducted using a randomized complete block design with four repetitions, in which treatments consisted of six nitrogen (N) topdressing doses (0, 50, 100, 200, 300, and 400 kg ha^−1^) during the 2022/2023 and 2023/2024 growing seasons. Evaluations of agronomic variables and image acquisition were performed in five distinct phenological stages throughout the maize crop cycle. The data were analyzed using deviance analysis and variance components, principal component analysis (PCA), and multivariate linear modeling for the prediction of field variables. Our results demonstrated that all indices were affected by the interaction between N doses and evaluation environments (phenological stages and growing seasons). Additionally, the most reliable were EXGRaw, TGI, GNDVI, NDRE, CIRE, GVI, CVI, BNDVI, PanNDVI, SRNIRRe, SFDVI, RGBindex, NDVI, SAVI, MSAVI, and OSAVI, which showed clustering patterns according to growing season condition and phenological stage. Finally, the variables predicted using the proposed methodology achieved coefficients of determination above 0.80, except for shoot biomass and 100-grain weight. Therefore, it can be concluded that vegetation indices are influenced by the evaluated environment; however, the proposed framework based on the deduction of fixed and random effects enables the prediction of field variables with high accuracy using relatively simple models.

## 1. Introduction

Plant phenotyping based on spectral information has become one of the most studied areas in plant science in recent years, aiming to quickly and non-destructively assess the morphophysiological condition of vegetation by capturing plant reflectance of plants in the visible and infrared regions of the electromagnetic spectrum [1,2,3]. In this way, plant phenomics has surpassed classical methods based on visual scoring, sampling, and weighing, which are time-consuming, resource-intensive, and do not meet the need to accelerate the release of new, more productive, and stable maize cultivars to address the global demand for food by 2050, especially in cereal production [4,5]. In this sense, maize traits directly associated with grain yield, such as number of grains per ear, weight of 1000 grains, and plant height, are very important and notoriously labor-intensive to measure [6].

Using multispectral or hyperspectral sensors, it has become possible to capture the electromagnetic energy reflected after interacting with the plant surface at wavelengths in the visible (400 to 700 nm) and infrared regions (750 to 2000 nm). This enables the early detection of physiological changes in vegetation caused by nutritional deficiencies, water shortage, or genotypic differences through spectral information [7,8,9]. Additionally, after obtaining the digital number for each wavelength from the images, different vegetation indices can be calculated and accurately correlated with plant growth, leaf area index, and chlorophyll content (r^2^ > 0.70) [10,11]. In this context, Zhou et al. [12] reported that spectral bands in the visible and near-infrared regions showed strong correlation with plant nitrogen concentration (r^2^ > 0.80), highlighting the potential use of lower-cost multispectral sensors for predicting the nutritional status of several grain crops.

Recently, plant phenomics has advanced toward high-throughput phenotyping, through the use of multispectral sensors embedded in unmanned aerial vehicles (UAVs) with greater spatial, spectral, radiometric, and temporal resolution. This approach allows faster data collection and higher image definition due to the smaller pixel size [13]. Furthermore, research has been conducted to adapt UAV-based field data collection for plant counting, inflorescence detection, characterization of crop growth curves, and assessment of genotype tolerance to stresses, enabling rapid evaluations throughout the entire crop cycle [14,15,16]. However, UAV-based phenotyping can be influenced by air temperature, light conditions, flight altitude above ground level, crop biomass accumulation, and treatment variability, which may affect the relationship between spectral information and field variables, often reducing prediction accuracy to coefficients of determination below 60% [8,17,18,19,20]. Moreover, the most effective vegetation indices for predicting field variables may vary depending on the evaluation environment, including genotype, phenological stage, and growing season [21].

Temporal analyses of vegetation indices can reveal patterns of plant growth and development, contributing to a better understanding of vegetation responses to environmental changes [22]. In this context, the methodology of mixed models enables the analysis of fixed and random effects that may influence data collection in experiments involving different genotypes, phenological stages, and growing seasons (years), based on restricted maximum likelihood (REML) and best linear unbiased prediction (BLUP) approaches [23]. This framework makes it possible to isolate specific effects, to determine parameter repeatability, and to estimate the error coefficient for each vegetation index in multiple scenarios [24,25]. Additionally, the interaction between flight timing (phenological stage) and years (growing season) is crucial to assess the reliability of spectral data for plant characterization and to select the best vegetation indices [21], aiming to identify stable indices for predicting agronomic variables. Therefore, the objectives of the present study were: (I) to evaluate the interaction of nitrogen doses and different evaluation environments (phenological stages and growing seasons), as well as the variance components associated with agronomic variables and vegetation indices, (II) to identify the best vegetation indices according to the evaluated environment, and (III) to predict agronomic variables in maize based on the relevant vegetation indices selected using the methodology proposed in our study.

## 2. Materials and Methods

### 2.1. Field Experiments and Experimental Design

**Figure 1:**
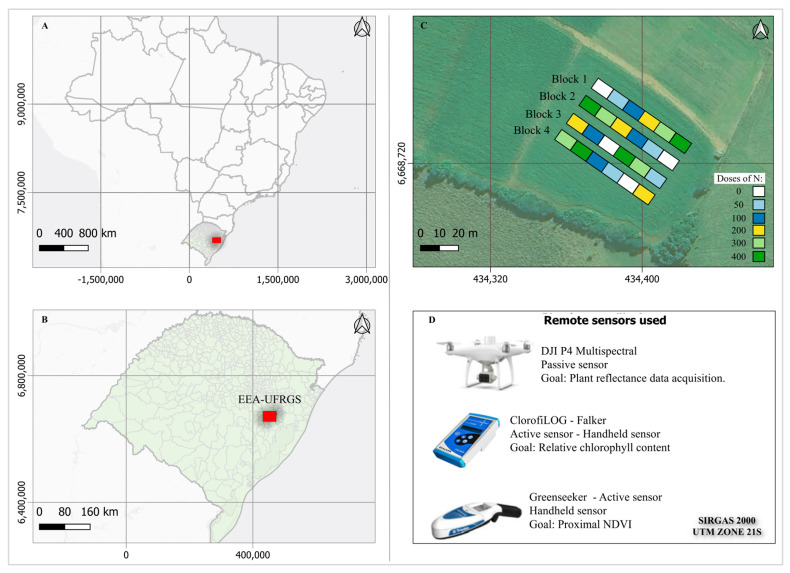
Localization of the experimental area (**A**,**B**), experimental design (**C**) and remote sensing platforms used (**D**).

**Figure 2:**
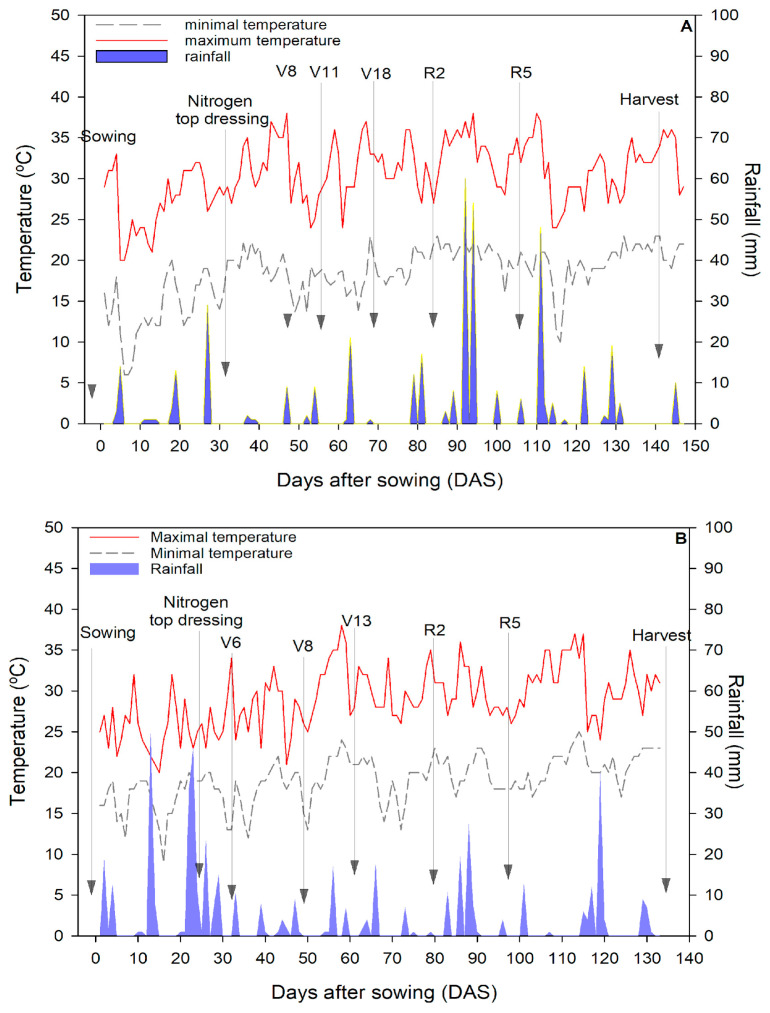
Temperature and precipitation data during the 2022/23 (**A**) and 2023/24 (**B**) growing seasons.

The study was conducted during the 2022/2023 and 2023/2024 growing seasons at the Agricultural Experimental Station of the Federal University of Rio Grande do Sul (UFRGS), located in Eldorado do Sul, Rio Grande do Sul State, southern Brazil (Figure 1A,B). The climate of the region is classified as Cfa (humid subtropical), characterized by average temperatures of 25 °C and regular rainfall distribution (Figure 2) [26,27].

A randomized complete block design was used, with four replications. Treatments consisting of six nitrogen (N) topdressing doses (0, 50, 100, 200, 300, and 400 kg ha^−1^) applied at the phenological stage of four fully developed leaves (growth stage V4) (Figure 1C).

In both growing seasons, the previous crop was oat (*Avena sativa*), which was terminated at flowering using Glyphosate (3 L ha^−1^) and Atrazine (4 L ha^−1^). Subsequently, the maize hybrid P3016 VYHR was sown under a no-tillage system at a density of 8 plants per m^2^ in the second half of October in both years. Each experimental plot consisted of five rows, five meters long each, with a row spacing of 0.50 m. At sowing, 20 kg ha^−1^ of N, 120 kg ha^−1^ of P_2_O_5_, and 60 kg ha^−1^ of K_2_O were applied in the sowing furrow. All other agronomic practices were carried out according to the technical recommendations for maize cultivation in southern Brazil.

### 2.2. Agronomic Variables Evaluated

Field evaluations were conducted at phenological stages V8 (eight fully developed leaves), V11, V18, R2 (onset of grain filling), and R5 (full dent stage) during the 2022/2023 season, and at V6, V8, V13, R2, and R5 during the 2023/2024 season. At each evaluation, plant height was measured in 10 plants per plot, from the soil surface to the last fully expanded leaf, using a graduated ruler, and expressed in meters. In addition, three plants per plot were cut, dried in a forced-air oven at 60 °C for determination of plant shoot dry biomass, expressed in kilograms of dry matter per hectare. Afterwards, the plant material was ground, and plant nitrogen concentration (PNC) was determined by the Kjeldahl method.

Portable proximal sensors were also used. Relative chlorophyll content (RCC) was measured at each phenological stage using a portable chlorophyll meter (Falker, Porto Alegre, Brazil) (Figure 1D). Measurements were taken on 10 plants per plot at the last fully developed leaf during the vegetative stage (season 2022/23: V8, V12, and V18; season 2023/24: V6, V8, and V13), and at the ear leaf during the reproductive stages (R2 and R5) in both seasons [28]. Additionally, proximal Normalized Difference Vegetation Index (NDVI) readings were obtained using the handheld Greenseeker sensor (Trimble, Westminster, CO, USA), equipped with an active light source emitting in the red (660 nm) and near-infrared (790 nm) regions, which estimates reflected radiation in these bands (Figure 1D). At the R6 stage (grain maturity), plants were harvested to determine the number of grains per ear (NGE), 100-grain weight (W100G), and grain yield. Grain yield was expressed in kilograms per hectare, at 13% grain moisture.

### 2.3. UAV-Based Image Acquisition and Vegetation Indices Calculation

**Figure 3:**
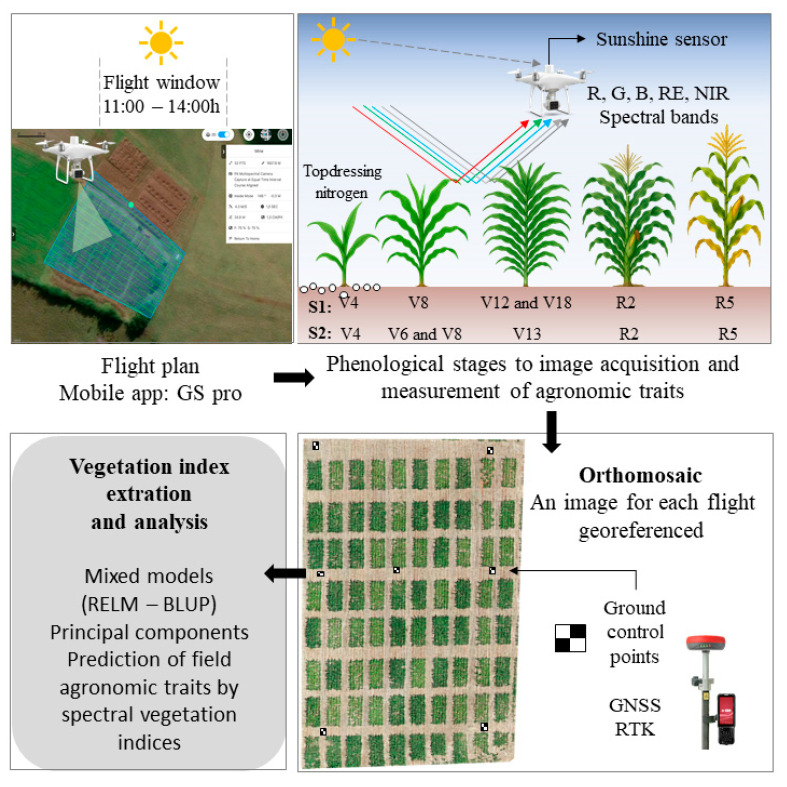
Workflow for data acquisition and analysis during the maize growing season of 2022/2023 (S1) and 2023/2024 (S2) in different phenological stages.

The collection of images for spectral evaluation of plots subjected to different N doses was carried out on the same day as the assessment of field variables, using a Phantom 4 Multispectral RTK drone (DJI, Shenzhen, China) (Figure 1D) and a pre-programmed flight plan with autonomous control via the GS Pro Mobile application (Figure 3). The multispectral sensor enabled the acquisition of plant reflectance in the red (R: 660 nm ± 16 nm), green (G: 550 nm ± 16 nm), blue (B: 450 nm ± 16 nm), red-Edge (RE: 730 nm ± 16 nm), and near-infrared (NIR: 840 nm ± 26 nm) bands (Figure 3). In addition, the sensor has a focal length of 5.74 mm, a pixel pitch of 6.1 μm and a ground sample distance (GSD) that varies according to flight altitude. Flight configurations consisted of 75% frontal and lateral overlap, a flight height of 30 m above ground level, and a nadir camera angle (90°). This configuration resulted in a spatial resolution of the orthomosaics of 3.2 cm pixel^−1^. All flights were conducted between 11:00 a.m. and 2:00 p.m. to ensure consistent illumination conditions. An onboard irradiance sensor (“sunshine sensor”) mounted on the upper surface of the drone was used to record solar irradiance for each spectral band during image acquisition. These measurements were subsequently used for radiometric correction and reflectance calibration of the images. Flights were conducted in “hover and capture at point” mode, in which the drone remains stationary during the acquisition of each image, eliminating motion blur and maximizing image sharpness, thereby ensuring higher image quality.

Image processing was performed using the software Agisoft Metashape (version 2.0.2) to generate the orthomosaics, which were georeferenced using seven ground control points measured with GNSS-RTK for accurate geometric correction. Subsequently, spectral information was extracted from the orthomosaics using the raster calculator tool in the software QGIS (version 3.40) to separate the spectral bands of each image. For each plot, the three central rows (4 m in length each) were delineated using a shapefile vector layer, and digital numbers were extracted for the red (R), green (G), blue (B), red-edge (RE), and near-infrared (NIR) bands. Finally, different vegetation indices were calculated using both visible (RGB) and multispectral bands (NIR-based) (Figure 3, Table 1).

### 2.4. Statistical Analysis

To evaluate the effects of N doses, evaluation environments (phenological stages × growing seasons), and their interaction, a restricted maximum likelihood (REML) approach was used to estimate variance components and obtain adjusted values for each agronomic variable. The significance of the interaction between factors was assessed through deviance analysis (*p* < 0.05) using the maximum likelihood ratio test (LRT). Subsequently, the following variance components and statistical parameters were estimated: phenotypic variance, repeatability coefficient (Rp), accuracy, and residual coefficient of variation (CVr). This procedure was based on the following statistical model:

Y{ij} = u + Bj + Di + Ej + eij

where (Y{ij}) is the observed value for each variable in each experimental plot; (u) is the overall mean; (Bj) represents the block effect; (Di) corresponds to the nitrogen dose effect; (Ej) represents the evaluation environment effect (phenological stage × growing season); and (eij) corresponds to the experimental error associated with each observation.

Based on the separation and quantification of these effects, adjusted values were obtained using the best linear unbiased predictor (BLUP). Thus, adjusted means were generated for each variable considering the variance structure associated with the evaluation environments. Prediction models and multivariate analyses were then developed using BLUP-adjusted values estimated for each nitrogen dose within each evaluation environment, where the evaluation environment factor represented the combination of growing season and phenological stage.

After identifying the vegetation indices with higher repeatability and lower residual variation, principal component analysis (PCA) was performed using the BLUP-adjusted values for each index. Grouping patterns of indices across evaluation environments were then considered in the interpretation of results.

To reduce redundancy among spectral variables and mitigate multicollinearity effects, the analytical pipeline combined variance component analysis, principal component analysis (PCA), and multivariate linear modeling. Only vegetation indices previously identified as stable and reliable through variance component analysis and PCA interpretation were considered for model development. The dataset was randomly partitioned into training (80%) and testing (20%) subsets. The training subset was used for model fitting and variable selection through backward stepwise regression (*p* < 0.05), whereas the testing subset was used to verify model consistency within the experimental dataset. Subsequently, multivariate linear models were fitted to evaluate the potential of vegetation indices to predict the following agronomic variables: NDVI (Greenseeker), relative chlorophyll content (RCC), shoot dry biomass, plant height, grain yield, number of grains per ear (NGE), 100-grain weight (W100G), and plant nitrogen concentration (PNC). Therefore, the coefficients of determination (R^2^) reported in this study should be interpreted as measures of model fit and predictive potential under the evaluated conditions rather than as evidence of model generalization to independent environments. All statistical analyses were performed in RStudio (version 4.3.1) using the EstimateBreed package [29].

## 3. Results

### Deviance and Variance Components Analysis

The different vegetation indices and agronomic variables were subjected to deviance analysis to evaluate the statistical influence of the factors “nitrogen doses” and “evaluation environments” (phenological stage and growing season) on the response of all variables analyzed in this study (Table 2). The deviance analysis using the RTL test revealed a significant effect for the Doses × Evaluation environments for all indices (*p* < 0.05), with no distinction between RGB and multispectral indices (Table 2). A similar result was observed for the field variables, except for the 100-grain weight (W100G), which showed a significant effect only for the applied nitrogen dose, with no influence from the evaluation environments (phenological stage and growing season). This finding indicates the stability of this variable even under contrasting environmental conditions.

The vegetation indices that showed adequate data quality were evaluated using the repeatability coefficient (Rp) and residual coefficient of variation (CVr) (Table 3). The vegetation indices EXGRaw, TGI, GNDVI, NDRE, CIRE, GVI, CVI, BNDVI, PanNDVI, SRNIRRe, and SFDVI presented high Rp values (>50%) and intermediate residual coefficients of variation (≤10%). In contrast, the indices RGBindex, NDVI, SAVI, MSAVI, and OSAVI showed lower Rp values (<40%); however, their residual coefficients of variation remained below 5%. Therefore, these indices demonstrate high potential for predicting agronomic variables due to the greater stability of the data across different evaluation environments. Additionally, the indices EXRRaw, RBI, and EVI were discarded because of their low data quality, characterized by Rp values < 10% and CVr values > 50%. Consequently, these indices were not considered in subsequent analyses.

RCC, plant height, and plant nitrogen concentration showed high Rp values and low residual coefficient, 60% and 5%, respectively (Table 3). Additionally, NDVI Greenseeker, NGE, W100G, and grain yield exhibited intermediate Rp values (40–50%), with a greater contribution from the error variation coefficient compared to the previously mentioned variables. In contrast, plant biomass presented a lower Rp (43%) and higher contribution from the residual factor (18%), due to variation in the evaluation environments tested in the experiment. Furthermore, the accuracy of all analyzed variables remained above 70% (Table 3). However, it is noted that the indices with higher Rp and lower residual variation coefficient achieved accuracy values above 95%. These results suggest that the experimental control was adequate, with a minor contribution of experimental error to the dataset.

**Figure 4:**
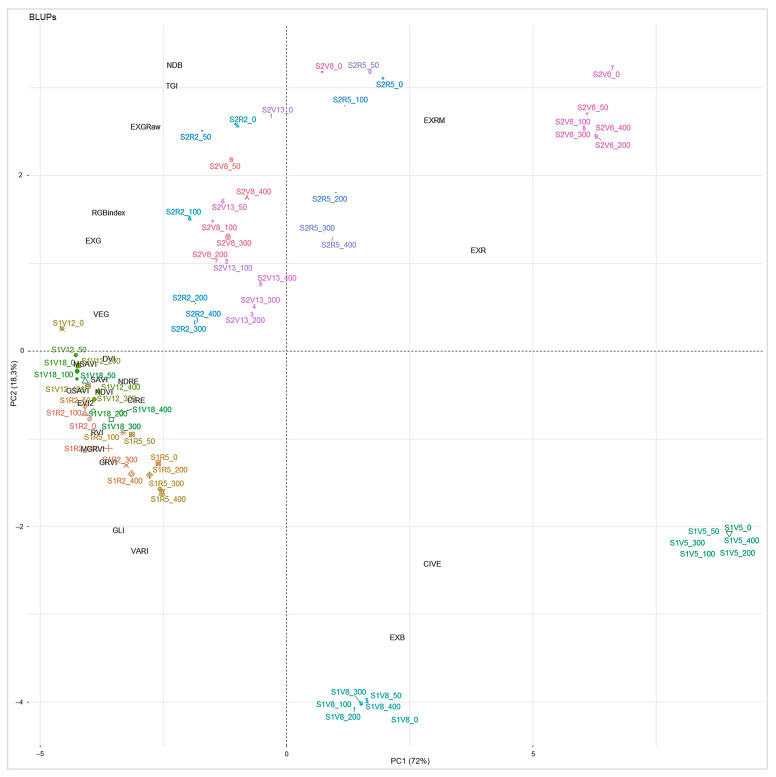
Principal components analysis of the BLUP values for each vegetation index. Variance explained by PC1 and PC2 was 72% and 18,3%, respectively.

After obtaining the BLUP values for each vegetation index (Supplementary Table S1), a principal component analysis (PCA) was performed using these values, enabling the identification of clusters associated with different nitrogen doses, phenological stages, and growing seasons. Overall, 90.3% of the variance was explained by the first two principal components plotted (Figure 4). The indices CIVE and EXB were more strongly associated with evaluation environments at early phenological stages (V5 and V8) during the 2022/23 growing season, indicating similar spectral behavior under conditions of lower canopy development. In addition, the indices GRVI, MGRVI, VARI, GLI, DVI, MSAVI, SAVI, NDRE, NDVI, OSAVI, CIRE, EVI 2, and RVI showed a consistent relationship with advanced phenological stages (V12, V18, R2, and R5), regardless of the nitrogen dose applied (Figure 4).

Similar to the behavior previously observed, differences among indices regarding phenological stages were also identified during the second growing season. Consequently, the indices grouped in 2023/2024 differed entirely from those observed in 2022/23. In this context, the PCA showed that the indices EXRM and EXR exhibited behavior similar to the variance observed exclusively at the V6 growth stage. On the other hand, NDB, TGI, EXGRaw, RGBindex, EXG, and VEG were grouped at the V8, V13, R2, and R5 phenological stages, regardless of the nitrogen dose applied (Figure 4). These results indicate that the specificity of the spectral parameters varies according to phenological stage and growing season, which may increase the complexity associated with the generalized use of phenotypic indices.

**Figure 5:**
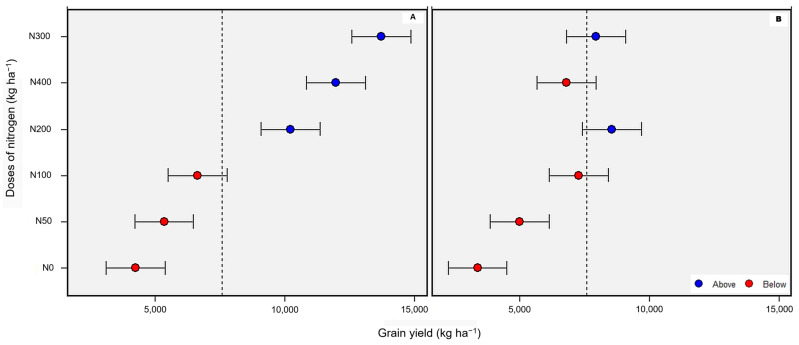
BLUP estimates of maize grain yield under different nitrogen doses during the 2022/2023 (**A**) and 2023/2024 (**B**) growing seasons.

Agronomic variables subjected to BLUP analysis showed that biometric parameters also differed according to nitrogen doses and evaluation environments (Supplementary Table S1). From the V12-V13 stage onwards, treatments receiving higher N doses (200, 300, and 400 kg ha^−1^) exhibited greater plant height, shoot biomass, relative chlorophyll content, and plant nitrogen concentration. A similar response was observed for grain yield components, achieving 475 grains per year in 2022/23 and 392 grains per year in 2023/24 at nitrogen doses of 300 and 200 kg ha^−1^, respectively. In addition, the highest grain yields were also obtained at these N doses, with values of 13,700 kg ha^−1^ and 8540 kg ha^−1^, respectively (Figure 5). These findings support the higher repeatability coefficients and lower residual coefficients observed in the variance component analysis for these parameters (Table 3), a pattern that was not clearly observed for NDVI Greenseeker and shoot biomass (Supplementary Table S1). Due to the specific sampling period (phenological stage) or growing season variability, some data showed CVr values > 10% or low Rp values, resulting in lower characterization efficiency of the evaluation environments. The weight of one hundred grains was not affected by the interaction between phenological stage and growing season, with variations associated only with nitrogen dose, reaching greater grain weight when plants received 400 kg N ha^−1^ (Supplementary Table S1).

**Figure 6:**
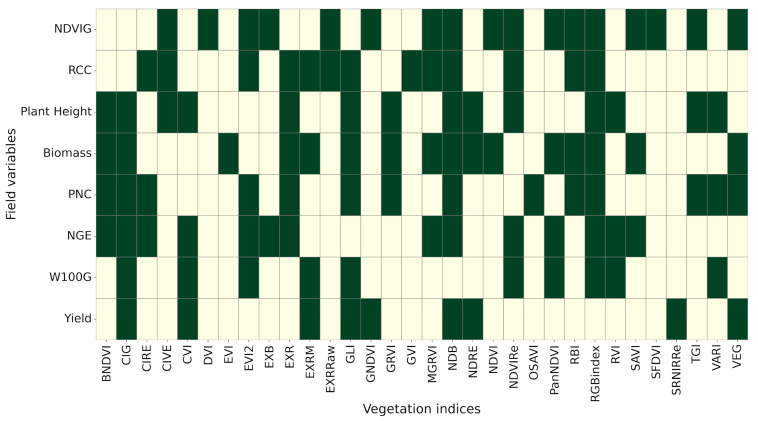
Map showing the frequency and number of predictor vegetation indices for each field variable in maize. Yellow and green rectangles represent the absence or presence, respectively, of each vegetation index in the prediction model for each variable.

Based on the responses observed for the different vegetation indices and agronomic variables, an attempt was made to establish prediction models for field variables using relevant spectral information through stepwise regression analysis. The models included the intercept of the multivariate linear regression and the angular coefficient of each vegetation index (Table 4). The adopted methodology enabled highly accurate predictions for most field variables (R^2^ > 0.80), except for shoot biomass and W100G, which exhibited lower coefficients of determination of 0.71 and 0.65, respectively. Furthermore, NDVI Greenseeker, relative chlorophyll content, plant height, shoot biomass, plant nitrogen concentration, and number of grains ear^−1^ required, on average, 15 vegetation indices within the prediction models (Table 4 and Figure 6). In contrast, W100G and grain yield required up to 10 predictors, highlighting the inherent complexity of each field variable and the need to identify relevant spectral vegetation indices under different evaluation environments.

From the synthesis of the different indices used in the prediction models (Figure 6), CIG, GLI, NDB, and RGBindex were able to predict 6 out of the 8 field variables evaluated. In addition, the indices BNDVI, CVI, EVI2, EXR, EXRM, MGRVI, NDVIRe, PanNDVI, RBI, RVI, and VEG were necessary to predict up to 5 field variables. Conversely, CIRE, CIVE, DVI, EVI, EXB, EXRRaw, GNDVI, GVI, NDRE, NDVI, OSAVI, RVI, SAVI, SFDVI, SRNIRRe, TGI, and VARI were associated with the prediction of 3 or fewer field variables. These findings demonstrate the existence of more generalist vegetation indices, whereas others exhibited a higher degree of specificity, depending on the variable considered.

## 4. Discussion

The modulation of maize response to nitrogen fertilization is influenced by environmental factors such as the phenological stage at which the application occurs, climatic conditions during the growing season, genotype, and the type of fertilizer applied [30,31]. Among these factors, interannual variation (growing seasons) can account for up to 50% of the response of the evaluated field variables, due to changes in rainfall availability and temperature range [32]. A similar pattern was observed in the present study, where seasonal variation resulted in changes of 10% to 30% in plant height, shoot biomass, and grain yield (Supplementary Table S1, Figure 5). These effects also altered the magnitude and behavior of vegetation indices, reinforcing the need to use large spectral datasets with greater temporal and spatial information to identify indices that are less susceptible to environmental variability [33].

The deviance analysis showed that all vegetation indices were affected by the interaction between nitrogen doses and evaluation environments (phenological stage and growing season) (Table 2). In this context, [21] reported that variation in vegetation indices collected at different flight dates can be explained by physiological and morphological changes expressed throughout plant development. These changes are associated with leaf structure, chemical composition, and plant-environment interaction, which alter light reflectance patterns and, consequently, the dynamics of vegetation indices [16]. In contrast, the weight of 100 grains was not influenced by the interaction between nitrogen doses and environments, which may be explained by a stronger genetic contribution, lower environmental sensitivity, and management-environment interaction governing the expression of this grain yield component, resulting in greater stability across environments [34,35,36]. Additionally, W100G was measured only after harvest, which reduces the influence of phenological stages on the variance of this field variable.

Restricted maximum likelihood (RELM) analysis is a method for estimating variance components in a dataset, enabling the identification of significant variables with high repeatability, reduced influence of experimental error, and greater reliability in prediction models [23,24].

In this context, repeatability coefficient analysis allows the identification of vegetation indices with greater stability and lower residual variation across nitrogen doses and evaluation environments. In addition, variables exhibiting higher repeatability and lower residual coefficients of variation demonstrate greater consistency and reliability for subsequent predictive modeling, particularly when they present Rp > 50% and low experimental error (CVr < 10%) [37,38]. In this sense, EXGRaw, TGI, GNDVI, NDRE, CIRE, GVI, CVI, BNDVI, PanNDVI, SRNIRRe, and SFDVI showed the highest repeatability values combined with acceptable residual variation in the present study. Similar responses were reported by [39], who identified these same stable spectral indices under contrasting environmental conditions using UAV-based multispectral phenotyping in soybean. Additionally, [33] emphasized that most vegetation indices are influenced by the flight timing (phenological stage) and experimental conditions, with greater environmental contributions and lower repeatability coefficients observed for EXG, GLI, MGRVI, RGBindex, and VARI. Consistent with the present results (Table 3), these indices were still considered relevant for phenotyping due to their low coefficient of experimental error. These findings corroborate with [40], who reported that EXG, GLI, and VARI were important for evaluating white oats using a spectral approach for lodging resistance, suggesting that vegetation index performance is highly specific to crop type and experimental condition. This behavior may be associated with the dominant wavelength characteristics at the time of image acquisition, as radiation availability, temperature, and flight timing can affect the ability of indices to properly distinguish differences among treatments [8]. Therefore, in the present study, UAV flights were conducted close to solar noon and preferably under clear-sky conditions, given that variations in illumination can significantly increase standard deviation and absolute error in predictions based on spectral information [19].

The PCA based on BLUP values revealed a clustering pattern among vegetation indices according to the evaluation environments (phenological stage and growing season) (Figure 4). The proposed approach incorporates the temporal structure of the crop cycle through the evaluation environment factor, thereby avoiding the direct pooling of spectral information from biologically distinct phenological stages without appropriate adjustment. The influence of the evaluation environment on index expression corroborates the findings of Shrestha et al. [18], who reported, over three years of maize trials, that the most effective vegetation index depended on both the phenological stage and climatic conditions of the growing season. Their results indicated that no single index consistently predicted grain yield accurately across all evaluation environments. This pattern was further supported by the PCA results, which showed clear stage-dependent clustering of vegetation indices across evaluation environments. Additionally, in both growing seasons evaluated in this study, the highest number of indices exhibiting similar behavior was identified at the later phenological stages (V12, V18, and R2) (Figure 4). This pattern is expected, given the limited differences among plants in terms of shoot biomass and soil cover during the early growth stages, as well as the reduced influence of the soil background in the imagery from V12 onward. [3] investigated the efficiency of predicting maize productivity throughout the crop cycle using spectral data collected by UAV, with flights conducted from V2 (two fully developed leaves) to physiological maturity (R6). These authors found that productivity prediction accuracy increased substantially (r^2^ > 0.80) from the V14 stage (fourteen fully developed leaves) onward. The highest predictive accuracy was achieved when data from the tasseling, full flowering, milk grain, and dough grain stages were combined (r^2^ > 0.93).

The analyzed environments also influenced the type of indices grouped together. Environments characterized by lower shoot biomass, plant height, relative chlorophyll content, number of grains per ear, and grain yield (Supplementary Table S1 and Figure 5) were associated with indices derived exclusively from the visible spectrum (CIVE, EXB, EXRM, EXR, NDB, TGI, EXGRaw, RGBindex, EXG, and VEG). This pattern was particularly evident during the 2023/24 growing season, which was marked by high rainfall volumes and a greater number of cloudy days, resulting in lower grain yields (<9000 kg ha^−1^) (Figure 2 and Figure 5). In contrast, the multispectral indices NDRE, CIRE, NDVI, SAVI, MSAVI, OSAVI, RVI, DVI, and EVI2 were grouped in the 2022/23 season, which exhibited greater shoot biomass, plant height, and grain yield (>14,000 kg ha^−1^), likely due to a more favorable rainfall distribution, temperatures close to 30 °C, and fewer cloudy days (Figure 2).

Studies comparing RGB- and multispectral-based indices have shown that, under contrasting fertilizer rates and canopy cover conditions, visible-spectrum indices tend to perform better in environments with lower biomass and leaf area, exhibiting strong correlations with grain yield [41,42]. Greater soil exposure during the early stages of crop development may limit the performance of multispectral indices, whereas indices based on visible bands, such as EXG, CIVE, GRVI, EXGRaw, and NDB, provide a more accurate characterization of maize growth up to 60 days after plant emergence [43,44]. Conversely, multispectral indices incorporating red-edge and near-infrared bands, such as EVI, SAVI, OSAVI, GNDVI, NDVI, and NDRE, are more effective in detecting differences in shoot biomass under conditions of complete canopy cover [45,46]. Therefore, the composition of the index clusters was strongly associated with the environmental conditions observed in each growing season, with a predominance of multispectral indices in 2022/23 and visible-spectrum indices in 2023/24.

The collection of biometric data in the field remains a common practice and represents the basis of conventional plant phenotyping for evaluating crop responses to specific management practices [47]. Although this approach is effective for assessing crop performance, it constitutes a major bottleneck in plant breeding and experimental research due to its low operational throughput and high labor requirements, highlighting the need for alternative approaches capable of accelerating these processes [48]. In this context, field traits with high repeatability coefficients and low residual variance, such as plant height and relative chlorophyll content (Table 2), can serve as valuable predictors in plant phenotyping. These traits are directly associated with biomass accumulation, leaf nitrogen concentration, lodging resistance, and grain yield, making them particularly relevant for crop evaluation and selection programs [49,50].

The prediction of maize grain yield and other agronomic traits using vegetation indices has been extensively investigated in recent years, driven by the need to monitor crop growth and development under varying environmental conditions, genotypes, nutritional management strategies, and irrigation regimes [19,51]. These studies have highlighted the often non-linear relationship between spectral variables and grain yield, emphasizing the need for advanced analytical approaches capable of capturing complex interactions among variables and improving prediction accuracy. Furthermore, the overfitting of complex predictive models developed from spectral datasets with limited temporal and spatial variability may restrict their robustness and broader applicability across different environments and growing conditions [52,53].

According to the methodology proposed in this study, satisfactory prediction accuracies (R^2^ > 0.80) were achieved for most of the agronomic variables evaluated using only the relevant vegetation indices selected by the multivariate regression model (Table 4). This approach enabled the development of specific prediction functions for each field trait by integrating data from all UAV flights and growing seasons, following the application of REML-BLUP procedures and the selection of significant vegetation indices through backward stepwise regression. Such a framework is essential for reducing variation associated with experimental error, identifying reliable phenotypic traits, mitigating the effects of multicollinearity, reducing the dimensionality of predictor variables, and selecting the most informative indices to compose robust prediction models [25,33,54].

Furthermore, indices such as CIG, GLI, NDB, and RGBindex were repeatedly selected across multiple predictive models, suggesting a broader applicability for estimating the agronomic traits evaluated in this study. In contrast, more specific indices, including NDVI, SAVI, GNDVI, NDRE, CIRE, GVI, CVI, PanNDVI, SRNIRRe, and SFDVI, exhibited higher angular coefficients within the prediction models (Table 4), indicating stronger associations with particular agronomic response variables [55]. These findings are consistent with the results of the variance component analysis, which revealed higher repeatability coefficients and lower residual variation for the corresponding traits (Table 3).

Among the predicted variables, W100G presented the lowest coefficient of determination (R^2^ = 0.65), despite exhibiting high stability across the evaluated environments. This comparatively lower prediction accuracy may be associated with the reduced phenotypic plasticity and limited variability of this trait among treatments, which can constrain the ability of vegetation indices to detect subtle differences through canopy reflectance. Consequently, even under stable experimental conditions, the spectral sensitivity for predicting W100G may remain relatively low, resulting in reduced predictive performance compared with other agronomic traits.

Although the proposed models demonstrated high coefficients of determination for most agronomic variables, the absence of independent validation datasets and cross-validation procedures warrants caution when interpreting their predictive performance and generalizability. Recent studies on UAV-based sensing and predictive modeling have emphasized the importance of external validation approaches to ensure model robustness, reliability, and transferability across diverse environmental conditions and independent datasets [56].

Various predictive modeling approaches have been evaluated over time, differing in both their mathematical structure (linear or nonlinear) and computational complexity. Among these approaches, Random Forest and Support Vector Machine algorithms have been widely validated as effective tools for predicting grain yield, nutrient concentration, and plant biomass, often achieving high predictive accuracies (R^2^ > 0.70) [57,58]. However, the performance and practical applicability of these models may be compromised when trained on datasets characterized by multicollinearity, substantial residual variation, and low data reliability. In addition, their greater computational complexity and reduced interpretability may limit their adoption in plant phenotyping applications.

Multiple linear regression models are widely used in agricultural remote sensing; however, they often exhibit reduced predictive accuracy applied to large volumes of spectral data [59]. Nevertheless, feature selection, dimensionality reduction, and multicollinearity handling techniques can substantially improve the performance of these simpler models, enabling better characterization of plant physiological status using regression models based on a limited set of predictors [60,61,62]. Accordingly, this study highlights the relevance of the proposed methodology for developing context-specific models tailored to the evaluated environments, using vegetation indices that provide relevant spectral information and can be readily integrated into predictive equations.

## 5. Conclusions

This study aimed to evaluate the effects of fixed factors and environmental conditions on the dynamics of vegetation indices used for predicting agronomic variables in maize, emphasizing the importance of considering these factors for the appropriate selection of predictor indices in modeling approaches. The main conclusions were as follows: all vegetation indices were influenced by nitrogen doses and evaluation environments (phenological stages and growing seasons), with higher repeatability coefficients and lower coefficients of error variation observed for EXGRaw, TGI, GNDVI, NDRE, CIRE, GVI, CVI, BNDVI, PanNDVI, SRNIRRe, and SFDVI. Furthermore, a clear specificity was observed between vegetation indices and phenological stages, with multispectral indices predominantly associated with conditions of greater plant height, shoot biomass (e.g., V12, V13, V18, R2, and R5), and grain yield. In contrast, RGB-based indices were mainly grouped in environments characterized by lower shoot biomass at early growth stages (e.g., V6 and V8) and in growing seasons with lower grain yield potential. Finally, the proposed methodology enabled the identification of the most suitable indices for estimating maize agronomic variables with high accuracy using a multivariate linear modeling approach.

## Figures and Tables

**Table 1:** List of spectral vegetation indices calculated in this study.

Type	Vegetation Indices	Equation
**RGB**	Excess of green (EXG)	2 ∗ [G/(R + G + B)] − [R/(R + G + B)] − [B/(R + G + B)]
	Excess of green raw (EXGRaw)	2 ∗ G − R − B
	Excess of red (EXR)	(1.4 ∗ R − G)/(R + G + B)
	Excess of red raw (ExRRaw)	1.4 ∗ R − G
	Vegetation extraction color index (CIVE)	(0.44 ∗ R−0.81 ∗ G + 0.39 ∗ B)/(R + G + B)+ 18.79
	Normalized green-red index (GRVI)	(G − R)/(G + R)
	Modified excess of red (ExRM)	(2 ∗ R − G − B)/(R + G + B)
	Vegetative index (VEG)	G/(r^0.667^ × b^0.333^) r = R/(R + G + B), g = G/(G + R + B), b = B/(R + G + B)
	Excess of blue (ExB)	(1.4 ∗ B − G)/(R + G + B)
	Normalized difference of blue (NDB)	(G − B)/(G + B)
	Atmospheric resistant index in the visible (VARI)	(G − R)/(G + R - B)
	Green leaf index GLI	((G - R) + (G - B))/(2 ∗ G + R + B)
	Triangular green index (TGI)	TGI = −0.5 × [190 (R − G) − 120(R − B)]
	Red-Blue index (RBI)	(R − B)/(R + B)
	Modified green-red index (MGRVI)	(G^2^ − R^2^)/(G^2^ + R^2^)
	RGB index RGBindex	(G^2^−B ∗ R)/ (G^2^ + B ∗ R)
**MULTIESPECTRAL**	Normalized Difference (NDVI)	(NIR – R)/(NIR + R)
	Red Edge (NDRE)	(NIR – RE)/(NIR + RE)
	Soil Adjusted Index (SAVI)	(1 + 0.5) ∗ [(NIR – R)/(NIR + R + 0.5)]
	Modified Soil Adjusted Index (MSAVI)	2 ∗ NIR + 1 − √(2 ∗ NIR + 1)^2^ − 8(NIR − R)]/2
	Optimized Soil Adjusted Index (OSAVI)	1.16 ∗ ((NIR−R)/(NIR + R + 0.16))
	Enhanced Vegetation Index (EVI)	2.5 ∗ (NIR-R)/(NIR + 6 ∗ R −7.5 ∗ B + 1)
	Normalized Difference of Green (GNDVI)	((NIR−G)/(NIR + G))
	Green Chlorophyll Index (CIG)	(NIR/G) − 1
	Rededge Chlorophyll Index (CIRE)	(NIR/RE) − 1
	Simple Ratio Index (RVI)	NIR/R
	Vegetation Difference Index (DVI)	NIR−R
	Green Vegetation Difference Index (GDVI)	NIR−G
	Green Ratio Vegetation Index (GVI)	NIR/G
	Vegetation-Chlorophyll Index (CVI)	(NIR ∗ R)/(G^2^)
	Enhanced Vegetation Index 2 (EVI2)	2.5 ∗ (NIR−R)/(NIR + 2.4 ∗ R + 1)
	Normalized Difference of Blue (BNDVI)	(NIR − B)/(NIR + B)
	Normalized Difference R-Re (NDVIRE)	(RE − R)/(RE + R)
	PanNDVI (PanNDVI)	(NIR − (G + R+B))/(NIR + (G + R+B))
	Simple Ratio NIR/RE (SRNIRRe)	NIR/RE
	Spectral Feature Depth (SFDVI)	((NIR + G)/2)/((R + RE)/2)

**Table 2:** Deviance analysis using the maximum likelihood ratio test (LRT) (*p* < 0.05).

**Model**	**EXG**	**EXGRaw**	**EXR**	**EXRRaw**	**CIVE**	**GRVI**	**EXRM**
Doses of N	5.24 × 10^−7^	2.21 × 10^−9^	0.00253	0.00057	2.46 × 10^−9^	0.00389	0.24621
Doses*Evaluation Environments	9.09 × 10^−5^	1.72 × 10^−19^	2.9 × 10^−11^	1.62 × 10^−16^	5.50 × 10^−19^	1.31 × 10^−14^	2.54 × 10^−21^
**Model**	**VEG**	**EXB**	**NDB**	**VARI**	**GLI**	**TGI**	**RBI**
Doses of N	5.36 × 10^−6^	0.00262	0.01216	0.00969	0.06862	2.43 × 10^−9^	1.00000
Doses*Evaluation Environments	4.60 × 10^−12^	6.69 × 10^−19^	2.9 × 10^−28^	3.62 × 10^−24^	5.35 × 10^−16^	9.89 × 10^−21^	1.73 × 10^−48^
**Model**	**MGRVI**	**RGBindex**	**NDVI**	**SAVI**	**MSAVI**	**OSAVI**	**EVI**
Doses of N	0.00514	4.18 × 10^−8^	2.3 x10^−7^	2.19 x10^−7^	9.61 × 10^−7^	2.32 x10^−7^	0.56113
Doses*Evaluation Environments	4.27 × 10^−8^	1.71 × 10^−5^	2.9 × 10^−10^	2.83 × 10^−10^	2.01 × 10^−8^	2.96 × 10^−10^	3.49 × 10^−7^
**Model**	**GNDVI**	**CIG**	**NDRE**	**CIRE**	**RVI**	**DVI**	**GDVI**
Doses of N	2.35 × 10^−14^	3.03 × 10^−12^	1.6 × 10^−13^	8.02 × 10^−12^	1.20 × 10^−10^	9.48 × 10^−12^	6.69 × 10^−14^
Doses*Evaluation Environments	4.86 × 10^−6^	3.88 × 10^−10^	1.5 × 10^−27^	5.06 × 10^−31^	5.99 × 10^−6^	3.65 × 10^−3^	4.34 × 10^−2^
**Model**	**GVI**	**CVI**	**EVI2**	**BNDVI**	**NDVIRe**	**PanNDVI**	**SRNIRRe**
Doses of N	3.03 × 10^−12^	1.76 × 10^−12^	5.8 × 10^−8^	2.62 × 10^−11^	8.48 × 10^−2^	1.58 × 10^−13^	1.58 × 10^−12^
Doses*Evaluation Environments	3.88 × 10^−10^	6.33 × 10^−14^	9.4 × 10^−11^	1.11 × 10^−5^	8.08 × 10^−6^	2.85 × 10^−5^	2.50 × 10^−19^
**Model**	**SFDVI**	**NDVIG**	**RCC**	**Plant height**	**Shoot biomass**	**NGE**	**W100G**
Doses of N	3.15 × 10^−14^	5.7 × 10^−8^	1.3 × 10^−9^	9.4 × 10^−11^	2.41 × 10^−6^	0.11902	0.04193
Doses*Evaluation Environments	2.43 × 10^−3^	4.3 × 10^−7^	1.7 × 10^−22^	3.8 × 10^−27^	5.22 × 10^−29^	1.5 × 10^−8^	0.24488
**Model**	**Grain yield**	**Plant nitrogen content (PNC)**					
Doses of N	0.13656	1.59 × 10^−12^					
Doses*Evaluation Environments	1.9 × 10^−8^	6.50 × 10^−13^					

NDVIG: NDVI Greenseeker, RCC: Relative chlorophyll content, Shoot biomass: Shoot dry biomass (kg ha^−1^), NGE: number of grains per ear, W100G: weight of one hundred grains, Plant nitrogen content (PNC): % of nitrogen in plant.

**Table 3:** Estimates of variance components by restricted maximum likelihood (REML) for the vegetation indices and variables analyzed in different phenological stages and two growing seasons in maize.

Variables	Phenotypic Variance	Repeatability Coefficient	Accuracy	CVr
EXG	<0.01	0.30	0.96	7.73
EXGRaw	34,022.94	0.50	0.97	10.50
EXR	<0.01	0.17	0.90	24.02
CIVE	5511.53	0.50	0.97	11.51
GRVI	<0.01	0.17	0.89	9.50
EXRM	<0.01	0.06	0.71	16.04
VEG	0.03	0.32	0.95	5.19
EXB	<0.01	0.20	0.89	12.73
NDB	<0.01	0.17	0.87	5.14
VARI	0.01	0.17	0.87	9.03
GLI	<0.01	0.10	0.81	2.64
TGI	8538.50	0.51	0.97	10.07
MGRVI	<0.01	0.14	0.88	8.40
RGBindex	<0.01	0.35	0.96	5.15
NDVI	<0.01	0.42	0.97	3.86
SAVI	<0.01	0.37	0.96	3.87
MSAVI	<0.01	0.33	0.95	2.68
OSAVI	<0.01	0.42	0.97	3.86
GNDVI	0.01	0.69	0.99	5.24
CIG	1.46	0.66	0.99	13.22
NDRE	<0.01	0.67	0.98	7.02
CIRE	0.05	0.62	0.98	10.11
RVI	3.13	0.46	0.98	11.96
DVI	279,301.62	0.45	0.98	9.34
GDVI	400,991.23	0.60	0.99	10.07
GVI	1.46	0.66	0.99	10.56
CVI	0.53	0.69	0.99	10.80
EVI2	0.02	0.40	0.96	5.02
BNDVI	<0.01	0.58	0.98	2.44
NDVIRe	<0.01	0.09	0.83	5.34
PanNDVI	0.01	0.65	0.99	12.29
SRNIRRe	0.07	0.71	0.99	4.53
SFDVI	56,236.85	0.64	0.99	9.73
NDVIG	<0.01	0.46	0.97	4.62
RCC	55.00	0.62	0.98	5.88
Plant height	361.61	0.63	0.98	4.25
Shoot biomass	1,045,457,500	0.44	0.95	18.00
Number of grains ear^−1^	10553.18	0.59	0.97	8.31
100-grain weight	16.06	0.55	0.99	8.26
Grain yield	10,186,376.14	0.57	0.97	11.43
Plant nitrogen content (PNC)	0.32	0.69	0.99	12.00

CVr: coefficient of variation in the residual.

**Table 4:** Multivariate models for predicting agronomic variables based on vegetation indices in maize.

Predicted Variable	Selected Vegetation Indices	R^2^ Adjusted	*p* < 0.05
NDVI Greenseeker * (Intercept: 6.38)	** (0.003) EXRRaw, (−0.03) CIVE, (−3.0) VEG, (17.36) EXB, (59.65) NDB, (−0.02) TGI, (−19.69) RBI, (3.94) MGRVI, (−24.47) RGBindex, (3730) NDVI, (−2481) SAVI, (−11.65) GNDVI, (0.0003) DVI, (−2.83) EVI2, (−3.30) NDVIRe, (9.73) PanNDVI, (−0.001) SFDVI	0.93	2 × 10^−16^
Relative chlorophyll content (RCC) (Intercept: 1450)	(5757) EXR, (−0.07) EXRRaw, (0.04) CIVE, (−4421) EXRM, (4529) NDB, (−4299) GLI, (−1189) RBI, (1473) MGRVI, (−1987) RGBindex, (−15.71) CIRE, (2.20) GVI, (84.64) EVI2, (−194) NDVIRe	0.86	2 × 10^−16^
Plant height (Intercept: 60910)	(117800) EXR, (1.68) CIVE, (74660) GRVI, (37530) NDB, (−6745) VARI, (92030) GLI, (0.97) TGI, (−26700) RGBindex, (−268.60) CIG, (2008) NDRE, (70.05) RVI, (191.60) CVI, (−3249) BNDVI, (2095) NDVIRe	0.89	2 × 10^−16^
Shoot biomass (Intercept: −28000000)	(25860000) EXR, (21930000) GRVI, (21510000) EXRM, (−503500) VEG, (19100000) NDB, (50270000) GLI, (−6657000) RBI, (1308000) MGRVI, (−15950000) RGBindex, (204500000) NDVI, (−136300000) SAVI, (−1122) EVI, (−2947) CIG, (44410) NDRE, (−285500) BNDVI, (158100) PanNDVI	0.71	2 × 10^−16^
Plant nitrogen concentration (PNC) (Intercept: 1667)	(−2975) EXR, (−1502) GRVI, (40.29) VEG, (−1683) NDB, (76.47) VARI, (−2635) GLI, (0.005) TGI, (344.10) RBI, (959) RGBindex, (28.50) OSAVI, (0.43) CIG, (−1.41) CIRE, (−17.94) EVI2, (24.63) BNDVI	0.87	2 × 10^−16^
Number of grains ear^−1^ (Intercept0: 95389)	(−300483) EXR, (231189) EXB, (−118205) MGRVI, (50691) RGBindex, (65919) SAVI, (6727) CIG, (−2683) CIRE, (−2773) RVI, (−4105) CVI, (−101759) EVI2, (−47033) BNDVI, (−17368) NDVIRe, (142005) PanNDVI, (125000) NDB	0.80	4 × 10^−10^
100-grain weight (Intercept: −7230)	(6518) EXRM, (−280) VARI, (13488) GLI, (−1478) RGBindex, (−118) CIG, (50) RVI, (84) CVI, (609) EVI2, (168) NDVIRe, (−1238) PanNDVI	0.65	1 × 10^−7^
Grain yield (Intercept: −4217772)	(4862062) EXRM, (320572) VEG, (−1559654) NDB, (8022900) GLI, (22051) GNDVI, (14563) CIG, (307968) NDRE, (−26282) CVI, (−70675) SRNIRRe	0.85	1 × 10^−14^

* Intercept value of the equation for each model, ** Angular coefficient for each vegetation index within the model.

## Data Availability

The data supporting the study findings are available on request from the corresponding authors. The data are not publicly available due to the authors’ plan to conduct a series of follow-up studies based on this dataset.

## Supplementary Materials

The following supporting information can be downloaded at: https://www.mdpi.com/article/10.3390/plants15121782/s1, Table_Supplementary_1. This table contains all vegetation indices and field variables with values adjusted using the RELM-BLUP methodology.
